# Complete surgical resection improves outcome in INRG high-risk patients with localized neuroblastoma older than 18 months

**DOI:** 10.1186/s12885-017-3493-0

**Published:** 2017-08-04

**Authors:** Janina Fischer, Alexandra Pohl, Ruth Volland, Barbara Hero, Martin Dübbers, Grigore Cernaianu, Frank Berthold, Dietrich von Schweinitz, Thorsten Simon

**Affiliations:** 1 Department of Pediatric Oncology and Hematology, University Children’s Hospital of Cologne, Kerpener Str. 62, 50924 Cologne, Germany; 2 Department of Pediatric Surgery, Dr. von Haunersches Children‘s Hospital, Munich, Germany; 3 Division of Pediatric Surgery, University Children‘s Hospital of Cologne, Cologne, Germany

**Keywords:** Surgical oncology, High-risk neuroblastoma, Localized neuroblastoma, Neuroblastoma surgery

## Abstract

**Background:**

Although several studies have been conducted on the role of surgery in localized neuroblastoma, the impact of surgical timing and extent of primary tumor resection on outcome in high-risk patients remains controversial.

**Methods:**

Patients from the German neuroblastoma trial NB97 with localized neuroblastoma INSS stage 1–3 age > 18 months were included for retrospective analysis. Imaging reports were reviewed by two independent physicians for Image Defined Risk Factors (IDRF). Operation notes and corresponding imaging reports were analyzed for surgical radicality. The extent of tumor resection was classified as complete resection (95–100%), gross total resection (90–95%), incomplete resection (50–90%), and biopsy (<50%) and correlated with local control rate and outcome. Patients were stratified according to the International Neuroblastoma Risk Group (INRG) staging system. Survival curves were estimated according to the method of Kaplan and Meier and compared by the log-rank test.

**Results:**

A total of 179 patients were included in this study. 77 patients underwent more than one primary tumor operation. After best surgery, 68.7% of patients achieved complete resection of the primary tumor, 16.8% gross total resection, 14.0% incomplete surgery, and 0.5% biopsy only. The cumulative complication rate was 20.3% and the surgery associated mortality rate was 1.1%. Image defined risk factors (IDRF) predicted the extent of resection. Patients with complete resection had a better local-progression-free survival (LPFS), event-free survival (EFS) and OS (overall survival) than the other groups. Subgroup analyses showed better EFS, LPFS and OS for patients with complete resection in INRG high-risk patients. Multivariable analyses revealed resection (complete vs. other), and *MYCN* (non-amplified vs. amplified) as independent prognostic factors for EFS, LPFS and OS.

**Conclusions:**

In patients with localized neuroblastoma age 18 months or older, especially in INRG high-risk patients harboring *MYCN* amplification, extended surgery of the primary tumor site improved local control rate and survival with an acceptable risk of complications.

## Background

Neuroblastoma is the most common solid extracranial malignancy of childhood and the most common malignant tumor in infants [1]. Despite the advances in multidrug therapy, surgery still plays a major role in treatment of neuroblastoma (NB) [2]. Children presenting with localized disease have an overall better prognosis, mainly depending on the degree of tumor resection [3]. Indeed, completely resected tumors (INSS stage 1) rarely relapse and do not require postoperative chemotherapy [4, 5]. There is, however, still a controversy regarding the value of radical surgery for extended local disease [2]. Biological and clinical prognostic markers may help to stratify risks and guide therapy in these patients, but prospective randomized trials with surgical endpoints are still missing. In 2008, the INRG group implemented a pretreatment risk stratification considering age, *MYCN* amplification, histological tumor grade, Image Defined Risk Factors and 11q deletion to classify NB patients into very low-, low-, intermediate- and high-risk patients [6]. In this study, we analyzed the impact of the extent of tumor resection on outcome of patients older than 18 months with localized non-metastatic NB aged, who were treated according to the German prospective clinical trial NB97.

## Methods

All patients registered by the German prospective clinical trial NB97 between November 1, 1996, and September 30, 2004, were included in this analysis when they met the following criteria: stage 1–3 neuroblastoma diagnosed according to the International Neuroblastoma Staging System criteria [7]; age at diagnosis >18 months but less than 21 years. Written informed consent was obtained from patients or their guardians for participation in the study design, data collection and treatment (Registration number: NCT00017225, ClinicalTrials.gov).

The NB97 trial was a randomized trial comparing ASCT (autologous stem cell transplantation) and oral maintenance chemotherapy in high-risk patients. According to the NB97 protocol, the patients with localized NB were prospectively stratified in NB97 high-risk, NB97 standard-risk or NB97 low-risk subgroups according to INSS stage, age, *MYCN*-Status and threatening symptoms. NB97 standard-risk patients received 4 cycles of chemotherapy after first surgery and a second resection when necessary whereas NB97 low-risk patients were observed for up to 12 months with examinations and staging every 6 weeks. NB97 high-risk patients were randomized and received ASCT or oral maintenance therapy. The trial protocol had been evaluated by the Institutional Ethical Boards of the University of Cologne and participating hospitals. All patients participated in the trial after informed consent and on voluntary basis. Trial protocol and results of the primary trial end point have been published before [8, 9]. The NB97 protocol also provided clear recommendations on timing and extent of tumor resection. For example, initial or delayed complete resection was advised when no vascular structures or adjacent organs were involved. Incomplete resection was acceptable to reduce the risk of acute complications and long-term organ impairments. Nephrectomy or insertion of vascular prostheses was discouraged. Therefore, complete initial resection was reserved only for patients with well-encapsulated primary tumors. All other patients were recommended to undergo second look operations after four to six cycles of induction chemotherapy. Moreover, radiation therapy of 40 Gy was advised for patients with unresectable residuals of the primary tumor present after induction chemotherapy. Finally, radiation doses less than 40 Gy were applied to protect adjacent structures with low radiation tolerance [10]. Data on extent and complications of resection were collected prospectively using case report forms.

For this analysis, imaging reports, operation notes and pathology reports were retrospectively reviewed by two independent experienced physicians, and discrepant results were clarified after repeated joint review of the patients’ files. In this study, we distinguished between two types of operations, as described before [11]. Briefly, first operation was the tumor operation performed before or within the first six cycles of induction chemotherapy, and best operation was the most extensive removal of primary tumor tissue done at any time during first-line therapy. For outcome analysis, the extent of resection was classified as follows: no operation or biopsy removing less than 50% of tumor tissue; incomplete resection of 50% to less than 90% of tumor volume present at the time of surgery; gross total resection removing more than 90% of the tumor; or complete resection without macroscopic postoperative tumor residuals. It was not possible to include the category of microscopic complete resection because neuroblastomas were rarely removed in toto, and therefore, the pathologist often received several tumor fragments, making confirmation of microscopic complete resection impossible. IDRF were not established in 1997, and therefore, they had to be assessed retrospectively by review of initial imaging and operation notes based on the definitions published by the European International Society of Pediatric Oncology Neuroblastoma Group [12] and the International Neuroblastoma Risk Group [13, 14]. During induction chemotherapy, re-staging, including magnetic resonance imaging of the primary tumor site, bone marrow assessment, and metaiodobenzylguanidine (MIBG) scintigraphy, was scheduled after four to six chemotherapy cycles. If progression or relapse was suspected, complete staging was necessary. Disease status and response to treatment were categorized as complete remission, very good partial remission, partial remission, stable disease, or progression according to the published International Neuroblastoma Response Criteria [7]. Pretreatment Risk Stratification was performed retrospectively according to INRG staging system [6] considering age, *MYCN* amplification, histological grade, IDRF and 11q deletion stratifying all patients into INRG very low-, INRG low-, INRG intermediate- and INRG high-risk. Histology was defined by INPC guidelines as described before [15].

### Statistical analysis

For statistical analysis, the IBM SPSS software (Version 24.0.0; Armonk, NY, IBM Corp.) and R version 3.3.1 were used. Proportions were compared by Fisher’s exact test. Survival curves were estimated according to the method of Kaplan and Meier and compared by the log-rank test. Event-free survival (EFS) time was calculated as the time from diagnosis to event or last examination if the patient had no event. Relapse, progression, and death from disease or surgery-related were regarded as events. Local progression–free survival (LPFS) was calculated from diagnosis to relapse or progression of the primary tumor site or last examination if the patient had no local recurrence. This means that patients who died from metastatic disease without progression at the primary tumor site were censored at the time of death for LPFS analysis. Overall survival (OS) was calculated as the time from diagnosis to death from disease or surgery-related or last examination if the patient survived. Multivariable backward selected Cox regression analyses were performed to analyze the prognostic value of the following factors: stage (1 or 2 vs. 3), extent of best resection (complete vs. other), *MYCN* status (amplified vs. not amplified) and IDRF (no vs. >1). The likelihood ratio test *p*-value for inclusion was *p* ≤ 0.05.

## Results

### Patients

**Fig. 1:**
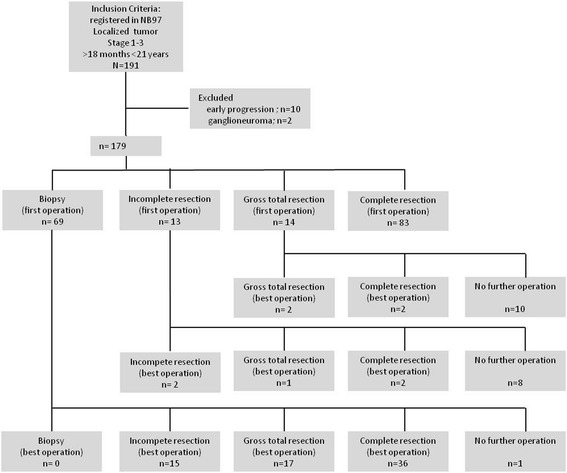
Patient flow diagram

Among 1121 patients in the NB97 trial, 191 patients with INSS stage 1–3 >18 months were eligible for analysis. Patients with INSS stage 4 disease (*n* = 383) and patients with non-stage 4 neuroblastoma <18 months of age (*n* = 547) did not meet inclusion criteria. Two patients with ganglioneuroma and 10 patients with an early progression within the first 40 days of chemotherapy were excluded from the study. 179 patients with localized neuroblastoma met all inclusion criteria (Fig. 1). The median age at diagnosis was 3.64 years (range, 1.5 to 20.8 years), and the median observation time of the surviving patients was 10.2 years (range, 0.5 to 17.3 years). 105 events were recorded. Among 30 relapses, there were 9 local relapses and in 35 patients with progression, we found 27 localized progressions of the primary tumor site. Thirty-three patients died. Table 1 lists details of the patients’ key characteristics.

**Table 1:** Key demographic and clinical characteristics of 179 patients with localized NB >18 months

Characteristic	No.	%
Sex		
male	86	48.0
female	93	52.0
Primary tumor site		
Neck	4	2.2
Chest	38	21.2
Abdomen	47	26.3
Adrenal	80	44.7
Pelvis	7	3.9
combined	3	1.7
MYCN status		
non-amplified	140	78.2
amplified	36	20.1
not available	3	1.7
INSS stage		
stage 1	68	38.0
stage 2	46	25.7
stage 3	65	36.3
NB97 risk group		
low	96	53.6
standard	36	20.1
high	47	26.1
IDRF		
yes	70	39.1
no	73	40.8
not available	36	20.1
INRG risk group		
very low	88	49.2
low	4	2.2
intermediate	23	12.8
high	36	20.1
not available	28	15.6
First operation		
biopsy only	69	38.5
incomplete resection	13	7.3
gross total resection	14	7.8
complete resection	83	46.4
Last operation		
biopsy only	0	0
incomplete resection	17	9.5
gross total resection	20	11.2
complete resection	40	22.3
Best operation		
biopsy only	1	0.6
incomplete resection	25	14.0
gross total resection	30	16.8
complete resection	123	68.7
Chemotherapy		
yes	82	45.8
no	97	54.2
RT of primary tumor site		
yes	7	3.9
no	172	96.1
myeloablative Chemo with ASCT		
yes	23	12.8
no	156	87.2

Before or during induction chemotherapy, complete resection was achieved in 83 patients (46.4%), gross total resection in 14 patients (7.8%), incomplete resection in 13 patients (7.3%), and biopsy in 69 patients (38.5%). For 8 patients no imaging reports were available and extent of resection was based on the case report forms of the trial only. During treatment, 77 patients had at least one more surgical resection of primary tumor site. After best surgery, 68.7% of patients achieved complete resection of the primary tumor, 16.8% gross total resection, 14.0% incomplete surgery, and 0.5% biopsy only.

The most radical resection of the primary tumor was achieved during first operation in 106 patients (59.2%) and during delayed surgery in 73 patients (40.8%). According to the protocol guidelines, 3.9% of all patients underwent external-beam radiation therapy of unresectable residuals of the primary tumor, in which case a median dose of 36 Gy was applied.

### Complications during first and best operation

A total of 56 patients experienced complications during 276 surgical interventions (20.3%) when undergoing first-line treatment for neuroblastoma. During first operation (*n* = 179) horner’s syndrome (3.9%), organ injury (3.4%) and intraoperative bleeding (2.8%) were the most frequent complications. No surgical related deaths occurred and the total complication rate was 19.6%. All other complications (infection, ileus, vessel or nerve injury) occurred in <1.7% of patients, respectively.

At best operation (*n* = 179) 50 complications in 40 patients occurred including horner’s syndrome with 5.6%, while organ injury and major bleeding/vessel injury account up to 4.4% and 3.9%, respectively. Two patients died due to surgery (1.1%). The complication rate at best surgery was 27.9%. Twenty-one patients (52.5%) out of 40 patients who experienced one or more complications had more than one operation.

The complication rate was not significantly different between patients with complete resection after best surgery (22.4%), patients with gross total resection (13.3%) and incomplete resection (32%; *p* = 0.395).

### Outcome after first operation

**Fig. 2:**
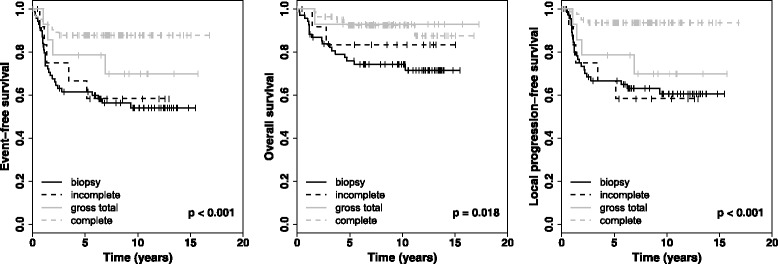
Kaplan-Meier-curves for all patients with localized NB (*n* = 179) after first operation

Complete resection could be achieved in 83/179 patients (46.4%). The 5-year EFS rate of all patients with complete resection was 87.8% ± 3.6%, the 5-year LPFS rate was 93.6% ± 2.7%, and the 5-year OS rate was 92.3% ± 3.0%. For patients with gross total resection, 5-year EFS and LPFS rate were 78.6% ± 11.0%, and the 5-year OS rate was 92.9% ± 6.9%. After incomplete resection, 5-year EFS and LPFS rate were 66.7% ± 13.6%, and the 5-year OS rate was 83.3% ± 10.8%. Patients with biopsy only had a 5-year EFS rate of 61.5% ± 5.9%, a 5-year LPFS rate of 66.7% ± 6.0%, and a 5-year OS rate of 75.8% ± 5.3% after first operation. The extent of first operation had an impact on EFS (*p* = 0.000), LPFS (*p* = 0.000), and OS (*p* = 0.018; Fig. 2; Table 2). Of note, any surgical resection compared to biopsy improved patient’s outcome in EFS and LPFS (*p* = 0.001) and OS (*p* = 0.002).

**Table 2:** Results of univariate outcome analysis of 179 patients with localized NB >18 months

	Biopsy (<50%)		Incomplete resection (50–90%)		Gross total resection (90–95%)		Complete resection (>95%)		*P* (comparing four groups)
Survival	Mean	SD	Mean	SD	Mean	SD	Mean	SD	
First operation									
No. of Patients	69		13		14		83		
5-year EFS	61.5	5.9	66.7	13.6	78.6	11.0	87.8	3.6	0.000
5-year OS	75.8	5.3	83.3	10.8	92.9	6.9	92.3	3.1	0.018
5-year LPFS	66.7	6.0	66.7	13.6	78.6	11.0	93.6	2.8	0.000
Best operation									
No. of Patients	1		25		30		123		
5-year EFS	censored	censored	58.0	10.2	59.8	9.0	82.8	3.4	0.001
5-year OS	censored	censored	70.5	9.4	75.4	8.1	90.8	2.7	0.020
5-year LPFS	censored	censored	66.2	10.4	62.7	9.0	87.1	3.1	0.001

### Outcome after best operation

**Fig. 3:**
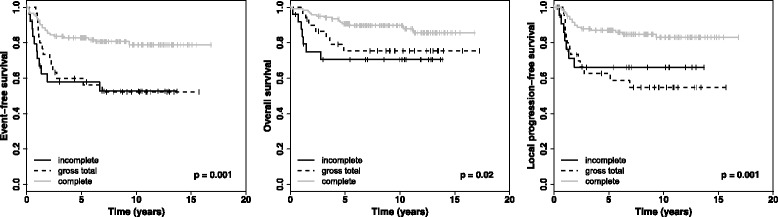
Kaplan-Meier-curves for all patients with localized NB (*n* = 179) after best operation

Complete resection could be achieved in 123/179 patients (68.7%). The extent of resection at the best operation was associated with better EFS (*p* = 0.001), better LPFS (*p* = 0.001), and better OS (*p* = 0.020). The 5-year EFS rate of patients with complete resection was 82.8.0% ± 3.4%, the 5-year LPFS rate was 87.1% ± 3.1%, and the 5-year OS rate was 90.8% ± 2.7%. For patients with gross total resection, 5-year EFS rate was 59.8% ± 9.0%, 5-year LPFS rate was 62.7% ± 9.0%, and the 5-year OS rate was 75.4% ± 8.1%. After incomplete resection, 5-year EFS rate was 58.0% ± 10.2%, 5-year LPFS rate was 66.2% ± 10.5%, and the 5-year OS rate was 70.5% ± 9.4%. One patient had a biopsy only and did not undergo a second operation, because he died eight months after diagnosis while undergoing ASCT. The 5-year survival rates are listed in Table 2, and life-tables are shown in Fig. 3. Multivariable analysis revealed resection (complete vs. other), and *MYCN* (non-amplified vs. amplified) as independent prognostic factors for EFS and OS.

### INSS stage and MYCN amplification

Subgroup analysis according to INSS stage after best operation confirmed all patients with stage 1 disease underwent complete surgical resection. In patients with INSS stage 2, there was no significant difference between incomplete resection, gross total resection and complete resection in EFS (*p* = 0.901), LPFS (*p* = 0.520) and OS (*p* = 0.446). But patients with INSS stage 3 disease showed a statistical significance according to the extent of resection in EFS (*p* = 0.032) and LPFS (*p* = 0.034), but not in OS (*p* = 0.053). Analyzing stage 3 patients with *MYCN* amplification (*n* = 29) and without *MYCN* amplification (*n* = 35) separately, *MYCN*-amplified patients showed a better outcome correlated to the extent of resection at best operation (EFS *p* = 0.001; LPFS *p* = 0.001; OS *p* = 0.002) whereas non-*MYCN*-amplified patients did not (EFS *p* = 0.411; LPFS *p* = 0.177; OS *p* = 0.905).

### INRG

According to INRG pretreatment risk classification 151 patients could be analyzed retrospectively. All patients with *MYCN* amplification belong to the high-risk group, resulting in 36 patients (20.1%). Patients with a histological category of GNB intermixed without *MYCN* amplification or IDRF (L1) are categorized as very low-risk (*n* = 88, 49.2%). Any patients with NB or nodular GNB harboring more than one IDRF (L2) may belong to low- (*n* = 4, 2.2%) or intermediate- risk (*n* = 23, 12.8%) depending on grade of tumor differentiation and 11 q aberration.

**Fig. 4:**
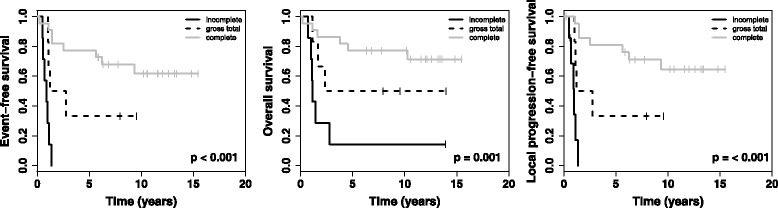
Kaplan-Meier-curves for INRG high-risk patients after best operation

High-risk patients (*n* = 36) show a better EFS (*p* = 0.001), LPFS (*p* = 0.001) and OS (*p* = 0.001) after best surgery when tumor resection was complete (Fig. 4). Patients belonging to intermediate-risk did not benefit from complete surgery (EFS *p* = 0.411; LPFS *p* = 0.177; OS *p* = 0.905).

## Discussion

In our cohort, extended surgery of the primary tumor site improved local control rate and outcome in patients older than 18 months with localized neuroblastoma. After complete resection during treatment the patients had a better local-progression-free survival (LPFS), event-free survival (EFS) and OS than the other groups. The wide range of clinical manifestations of neuroblastomas, their localization and their resectability leads to a high heterogeneity of our cohort. The NB97 protocol clearly recommended less aggressive surgical approach particularly during first operation in an effort to avoid surgical complications. Most surgeons choose a less aggressive approach when the disease was considered to be unresectable during operation. Thus, the question whether patients did not undergo complete resection because of less aggressive surgical approaches, or simply due to unresectable disease cannot be answered retrospectively. Moreover, less aggressive surgeons are more likely to classify patients as unresectable and vice versa. Prospective studies with a statement of the surgeon’s decision in his/hers operation notes are needed to address different approaches.

However, subgroup analyses showed better EFS and OS for patients with complete resection in INSS stage 3 and *MYCN* amplification. Patients with *MYCN* amplification belong to the INRG high-risk stratification and are therefore of special interest since the role of surgery in high-risk patients remains controversial. Other studies of high-risk patients especially evaluating stage 4 patients show no advantage of complete resection on patient’s outcome [11, 16], while lower local recurrence rates and better OS are reported after extended surgery by La Quaglia et al. [17, 18]. But analyzing localized neuroblastoma in patients >1 year, radical surgery is recommended which is in line with our findings [2]. A meta-analysis of 2599 patients with stage 3 and 4 neuroblastomas from 33 studies demonstrated that relative risk of mortality was decreased in patients who underwent >90% resection [19]. Our study indicates that complete surgical resection of the primary tumor site should be attempted in INRG high-risk patients.

Risk of complications at first and best operation is comparable to previously published data [2]. Since the presence of IDRF predicts the risk of complications and the extent of surgery, the impact of IDRF should become an integrated part of therapy planning as shown before [20–22], but cannot be safely used as an independent risk factor for outcome. Therefore, INRG classification considering IDRF, histological category, *MYCN* amplification, grade of tumor differentiation and chromosomal aberrations, was applied in our study and showed a significant difference in high-risk patients with complete surgical resection of the tumor. Future trials should encourage a complete resection of the primary tumor in INRG high-risk patients with a more aggressive approach to improve outcome with an acceptable risk of complication, but probable life-threatening complications should still be avoided.

In this study postoperative staging to assess the presence and extent of possible residual tumors was carried out by computed tomography, magnetic resonance imaging or ultrasound, but MRI was recommended as gold standard for follow-up. The interval between surgery and imaging varied between one and three months postoperatively, which can result in differences of the extent of resection described by imaging studies and by operation notes. Whenever there was inconsistency (26.7% of all cases), joint review with a radiologist was performed.

Even in patients with localized neuroblastoma older than 18 months, spontaneous regression or differentiation can occur at any time throughout therapy. This should be considered in patients with incomplete resection or gross total resection achieved at first operation. In this study, only few patients with incomplete/gross total resection after first operation underwent second operations to achieve complete resection, whereas most patients did not (9 vs. 18). Due to the small number of patients, a benefit of further surgery in these cases remains unclear. Possibly, molecular characterization of the primary tumors will be able to answer these questions in the future.

## Conclusion

In patients with localized neuroblastoma >18 months, especially with INRG high-risk classification, extended surgery improves EFS, LPFS and OS and should therefore be attempted.

## Abbreviations

ASCT: autologous stem cell transplantation
EFS: event-free survival
GNB: Ganglioneuroblastoma
Gy: Gray
IDRF: image defined risk factor
INPC: International Neuroblastoma Pathology Classification
INRG: International Neuroblastoma Risk Group
INSS: International Neuroblastoma Staging System
LPFS: local-progression-free survival
MIBG: metaiodobenzylguanidine scintigraphy
MRI: magnetic resonance imaging
MYCN: oncogene
NB: neuroblastoma
OS: overall survival
RT: radiotherapy
vs.: versus
